# A proposed convergent molecular chain linking senescence, cancer, and chronic infection to a correctable failure of dendritic cell instruction

**DOI:** 10.3389/fimmu.2026.1861044

**Published:** 2026-07-31

**Authors:** Andrew Caravello, Andrew Blidy

**Affiliations:** 1 Department of Emergency Medicine, Rowan-Virtua School of Osteopathic Medicine, Stratford, NJ, United States; 2 SaltusBiotech, Pleasanton, CA, United States

**Keywords:** dendritic cell, DunedinPACE, IL-12, immune surveillance, immunosenescence, IRF8, SASP, senescence

## Abstract

**Background:**

Three convergent phenomena in biomedical research have lacked a unified mechanistic explanation: the dendritic cell dysfunction of aging, the immune evasion of cancer, and the tolerogenic bias of chronic infection. Each is characterized by failure of type 1 conventional dendritic cell (cDC1) instruction and loss of its bioactive IL-12p70 output.

**Hypothesis:**

We propose that these three phenomena share a single molecular cause: sustained silencing of IRF8 by a four-step chain. SASP cytokines activate STAT3 in hematopoietic progenitors; STAT3 recruits DNMT1 and EZH2 to the IRF8 locus; dual DNA and histone methylation installs a bistable silencing state with no stable intermediate; IL-12 transcription and cDC1 instruction collapse. Each step is individually established in the literature. Their assembly is a deduction rather than a new discovery.

**Proposed correction:**

A cDC1 manufactured ex vivo outside the SASP-STAT3 field, the α-type-1 polarized dendritic cell (α-DC1), is not subject to the chain and produces IL-12p70 at supraphysiological concentrations. If the chain is correct, α-DC1 administration should restore endogenous IRF8 expression, re-enable cDC1 surveillance, and decelerate biological aging as measured by DunedinPACE. We outline the mechanism, testable predictions, and falsification criteria.

## 1 Introduction

Three of the most consistent findings in modern immunology share a common phenotype without a common explanation. Aged immune systems show weaker type 1 dendritic cell instruction and clear fewer senescent cells than young systems (1, 2, 52, 59, 60, 68). Tumor-infiltrating dendritic cells are skewed toward tolerogenic phenotypes and fail to license cytotoxic T cell responses (3, 4, 67), within a surrounding myeloid compartment biased toward suppressive, myeloid-derived suppressor cell-dominated states (8). Chronic viral infections and persistent intracellular bacterial infections are associated with dendritic cell exhaustion and attenuated IL-12 output (5, 6).

In each setting, the specific cell responsible for instructing effector immunity, the type 1 conventional dendritic cell (cDC1), is numerically depleted, functionally silent, or both (4, 7).

The parallel between these three settings has been noted but not explained at a mechanistic level. Existing models treat them as distinct processes that happen to share an endpoint.

Despite extensive study of each phenomenon in isolation, no unifying molecular mechanism has been proposed that accounts for the shared loss of cDC1 instruction across all three contexts.

The cDC1 is defined at the lineage level by its dependence on the transcription factor IRF8, which is required for cDC1 specification, survival, and the capacity to produce IL-12p70 (9, 10). The IRF8 locus is regulated by a BATF3-dependent enhancer architecture that Murphy and colleagues have shown to operate as a bistable switch with no stable intermediate state (11, 12): a committed cDC1 is either IRF8- high and surveillance-competent or IRF8-low and tolerogenic. The switch does not sit between these states. This architectural property has not been integrated into models of aged, tumoral, or chronically infected cDC1 failure, but it is central to understanding why partial interventions have produced only partial results.

We propose that aging, cancer, and chronic infection converge on a single molecular chain that forces this switch into the off position. The chain has four steps, each of which is individually established in the published literature. If the assembly is correct, three implications follow. First, the three clinical phenomena are not coincidentally similar; they are one phenomenon expressed in three contexts. Second, pharmacological interventions targeting single nodes of the chain, STAT3 inhibitors, DNMT inhibitors, EZH2 inhibitors, IL-6 blockade, cannot produce durable correction because remaining nodes continue to reinforce silencing. Third, there exists a specific therapeutic logic that bypasses rather than unlocks the architecture: a cDC1 manufactured outside the chain cannot be silenced by it, and should therefore restore the instruction its endogenous counterparts no longer provide.

We present the five premises underlying the chain, the convergence that follows from their assembly, the therapeutic corollary, and four testable predictions with explicit falsification criteria. The framework does not claim novelty at the level of individual molecular observations. It claims that the assembly of those observations, and the therapeutic prediction that follows from the assembly, are new.

## 2 The silencing chain

The chain has four steps across five nodes, and it rests on five premises, each established independently in peer-reviewed literature. The framework proposed here is the assembly of these premises into a single convergent mechanism: if each premise holds, the conclusion follows. We state each premise, cite its supporting literature, and then show how they combine.

### 2.1 Premise 1: IRF8 is necessary for cDC1 identity and IL-12 production

IRF8 is the master regulator of cDC1 lineage commitment.

Loss-of-function alleles eliminate the cDC1 compartment in mouse and human (9, 10, 13); forced expression directs progenitors into the cDC1 program.

IRF8 (ICSBP) is recruited to the IL12B (p40) promoter through protein-protein interaction at the Ets site and, together with IRF1, activates its transcription in macrophages (14).

This is an identity-level requirement, not a modulatory one.

IRF8 is nonetheless not the exclusive transcriptional input to IL-12: the IL12B (p40) promoter is combinatorial, occupied by NF-κB family factors and IRF1 alongside IRF8.

Its non-redundancy lies elsewhere.

Because IRF8 is required for the cDC1 to exist at all, redundancy at the IL-12 promoter is moot, since the cell that would fire it is never built.

And the bioactive IL-12p70 heterodimer requires the p35 subunit, which IRF8 co-activates with IRF1 (79), whereas the broadly regulated p40 subunit is shared with IL-23 and yields no bioactive cytokine alone.

The cDC1 is in turn the non-redundant *in vivo* source of the IL-12 that drives type-1 immunity (80).

IRF8 is therefore the single node at which the identity of the non-redundant IL-12 source and the gating of its bioactive output coincide, so a STAT3-dominated environment that silences this one locus removes the producer and its product together.

This is the structural reason a single lesion at IRF8 would be convergent across senescence, cancer, and chronic infection rather than specific to any one of them.

The dependence extends to the cDC1 lineage program: IRF8 is required for cDC1 development and lineage commitment, and its loss is not compensated by other maturation signals.

Any intervention that aims to restore cDC1 function without first restoring IRF8 expression is therefore constrained to working with a population whose foundational transcription factor is absent.

### 2.2 Premise 2: the IRF8 locus is bistable, not graded

The Murphy laboratory has mapped a +32 kb enhancer that, together with BATF3-dependent autoactivation, produces a switch-like expression pattern at the IRF8 locus (11, 12, 15).

The enhancer uses low-affinity AICE (AP-1–IRF composite element) binding sites whose suboptimal affinities are the topological basis for binary output: at threshold the enhancer is on, below threshold it is off, and no concentration of transcription factor produces a stable intermediate (16).

Bagadia and colleagues have further demonstrated an NFIL3-Zeb2-Id2 pathway that enforces the switch by imposing enhancer switching at the moment of cDC1 commitment (12).

This matters for the present argument because it means that partial correction of upstream signaling is insufficient to restore cDC1 function.

The locus is either open or closed.

A cDC1 that has flipped to the off configuration cannot be returned to the on configuration by gradual normalization of inputs; it requires either the complete removal of silencing signals or a bypass strategy that generates new cDC1s outside the silencing environment.

### 2.3 Premise 3: sustained STAT3 activation recruits DNMT and EZH2 to target promoters

Phosphorylated STAT3, when sustained rather than pulsatile, is acetylated at K685 by p300/CBP (17).

Acetylated STAT3 physically interacts with DNMT1 and directs it to target promoters (18, 19) where sustained STAT3 produced promoter hypermethylation and gene silencing (18) that could be reversed by resveratrol-induced STAT3 deacetylation (19).

Sustained STAT3 is proposed to also engage the Polycomb Repressive Complex 2 (PRC2), whose catalytic subunit EZH2 deposits H3K27me3 (20, 21). In glioblastoma stem-like cells, phosphorylated EZH2 methylates STAT3, enhancing STAT3 tyrosine phosphorylation and creating a feed-forward amplification loop that operates preferentially in stem-like cells (40). The STAT3-EZH2 interaction has been confirmed in independent cancer systems, including ovarian carcinoma where protein kinase A-mediated EZH2 phosphorylation regulates STAT3 activity (70), and in models where cytoplasmic EZH2 promotes JAK2-dependent STAT3 activation (71).

The dual-mark installation, DNA methylation by DNMT1 and histone methylation by EZH2, is the canonical signature of long-term gene silencing in vertebrate cells (22).

SOCS3 is frequently silenced by promoter hypermethylation, which relieves a principal negative feedback brake on JAK-STAT3 signaling (23).

PTPN1, a phosphatase that directly dephosphorylates STAT3, is recurrently inactivated by somatic coding-sequence mutations in STAT3-driven lymphomas (24).

CREBBP inactivation in germinal center B cells similarly expands the germinal center B cell compartment while down-regulating MHC II expression, promoting immune evasion (39). This route is not proposed as the sole or dominant mechanism of IRF8 repression: STAT3 also restrains cDC1 function through the JAK2 to STAT5 axis (74), and the two routes operate in parallel. The suppressive role of STAT3 has been demonstrated within cDC1 directly, where IL-10-activated STAT3 suppresses interferon beta, interferon gamma, STAT1 phosphorylation, and interferon-stimulated gene expression (75).

### 2.4 Premise 4: the IRF8 locus acquires DNA methylation and H3K27me3 in tolerogenic states

Bisulfite sequencing of tolerogenic dendritic cells and of dendritic cells from aged and tumor-bearing hosts shows methylation of CpG islands at the IRF8 promoter (25, 26).

Chromatin accessibility profiling in the same settings shows these regions colocalize with accessible distal enhancer elements, and cytokine signaling drives demethylation dynamics at dendritic cell identity loci (27, 28).

Loss of IRF8 transcription correlates with the acquisition of repressive marks (29).

Direct evidence connects the upstream signal to this mark, to the cooperating histone modification, and to its reversibility. IL-10-activated STAT3 binds the Dnmt1 and Dnmt3b promoters and drives IRF8 promoter hypermethylation in myeloid-derived suppressor cells (73); EZH2 is recruited to the IRF8 promoter to deposit H3K27me3 and downregulate IRF8, cooperatively with DNA methylation (83); DNA methylation of the IRF8 promoter represses IFN-γ-induced, STAT1-mediated IRF8 activation in colon carcinoma cells (81); and demethylation with decitabine reverses IRF8 promoter hypermethylation and restores endogenous IRF8 expression, in multiple myeloma (82) and in myeloid-derived suppressor cells driven by autocrine IL-6 through the same STAT3 axis (77). The same axis that installs the dual mark also gates its interferon-driven reversal.

Demethylating agents such as 5-aza-2′-deoxycytidine inhibit DNA methylation and can reactivate epigenetically silenced genes (30); in these models they would be predicted to only partially restore IRF8, consistent with the dual-mark architecture requiring both DNA and histone demethylation.

Pacis and colleagues have shown that bacterial infection specifically remodels the DNA methylation landscape of human dendritic cells, establishing that the methylation state is environmentally induced rather than constitutive (27).

Age-related methylation changes at immunity loci have been documented in humans by Tserel and others (25).

The methylation signature is therefore not theoretical; it has been directly observed in the three clinical contexts, aging, cancer, and chronic infection, that the framework proposes to unify. The one context in which the measurement in cDC1 specifically remains to be made, the aged human cDC1, is filed as Prediction 1 with an explicit falsification criterion (**Table 1**).

**Table 1:** Four testable predictions of the framework with explicit falsification criteria.

#	Prediction	Falsification criterion
1	IRF8 promoter and +32 kb enhancer methylation increase with biological age and decrease following α-DC1 vaccination in peripheral blood cDC1s.	Bisulfite sequencing of cDC1s from α-DC1-vaccinated subjects shows no reduction in IRF8 methylation relative to baseline at 12 weeks.
2	IL-12p70 production capacity of peripheral cDC1s recovers toward young-adult levels within 6 months following α-DC1 vaccination.	Ex vivo stimulated cDC1s from α-DC1-vaccinated subjects do not show increased IL-12p70 production at 6 months.
3	Senescent cell burden (p16INK4a transcript and SA-β-gal staining) decreases in peripheral tissues following α-DC1 vaccination.	Senescent cell burden does not decrease by 6 months in α-DC1-vaccinated subjects.
4	DunedinPACE deceleration follows α-DC1 vaccination, with the magnitude proportional to cDC1 recovery.	DunedinPACE shows no deceleration at 12 months following α-DC1 vaccination.

### 2.5 Premise 5: SASP cytokines sustain STAT3 activation in hematopoietic progenitors

Senescent cells (53, 54, 56, 65) secrete a characteristic cytokine profile (SASP) dominated by IL-6, IL-1β, TNF-α, and TGF-β, with additional contributions from PGE2, CXCL12, and amphiregulin (31–34).

Chronic IL-6, in particular via trans-signaling through soluble IL-6 receptor, produces sustained rather than pulsatile STAT3 activation (35). The distinction between pulsatile and sustained STAT3 is critical: pulsatile signaling activates transient gene expression programs that return to baseline, while sustained signaling produces the chromatin-level consequences described in Premise 3.

Doolittle and colleagues review evidence that the aged bone marrow microenvironment accumulates senescent osteoprogenitors, osteocytes, and myeloid cells whose SASP shifts the local environment toward reduced bone formation and increased resorption (36).

Kovtonyuk and colleagues have documented the broader phenomenon of inflamm-aging of hematopoiesis, in which chronic low-grade inflammation from both SASP and other sources shifts progenitor output toward myelomonocytic fates at the expense of lymphoid commitment (38). The senescent marrow environment is causally implicated in progenitor dysfunction, since clearance of senescent cells rejuvenates aged hematopoietic stem cells (37, 61). Given the IL-6 trans-signaling biology of Premise 3, this SASP-rich niche is expected to impose sustained rather than pulsatile STAT3 activation on the progenitors that transit it.

Crucially, the inducing environment is not limited to aged tissues. Tumor stromal senescence produces a SASP-like paracrine field that exposes cDC1 progenitors to the same signaling pattern (32, 33, 66). For chronic infection, the most direct evidence is at the level of the dendritic-cell methylome, where bacterial infection remodels DNA methylation at identity loci [Premise 4 (27)]; whether a SASP-STAT3 field is the dominant driver in that setting, rather than other STAT3 activators, remains to be quantified (Section 5).

### 2.6 Deduction: the convergent chain

**Figure 1:**
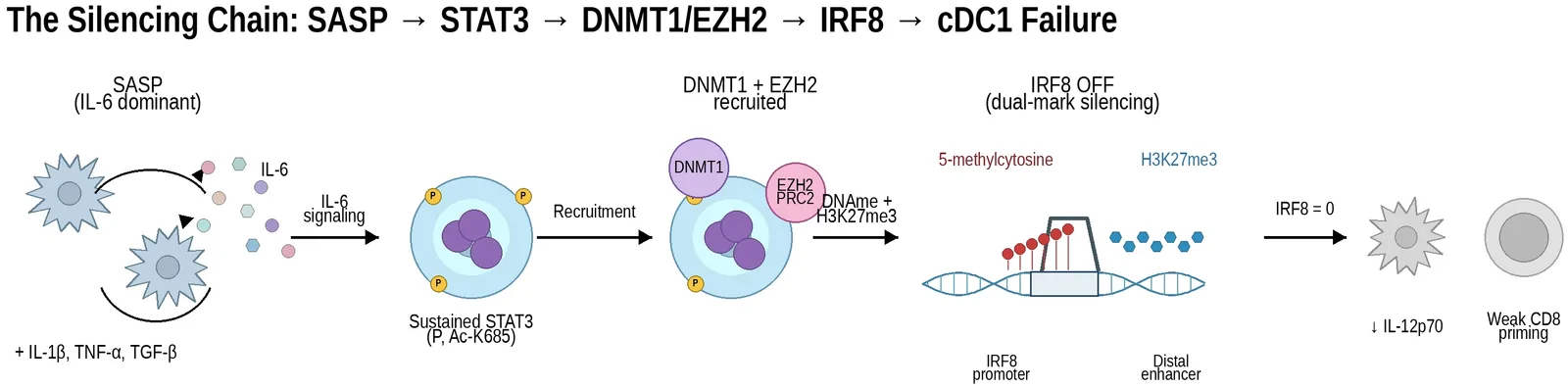
The silencing chain. Four-step molecular chain (five nodes) from SASP cytokines through STAT3 to IRF8 silencing and cDC1 instruction failure. Each arrow represents an established molecular relationship supported by cited primary literature. The bistable switch at the IRF8 locus, shown in the OFF position, has no stable intermediate state. Dual DNA methylation (lollipops on promoter) and H3K27me3 (hexagons on +32 kb enhancer) installation is shown.

When Premises 1 through 5 are assembled, the following conclusion follows. SASP cytokines sustain STAT3 in cDC1 progenitors (Premise 5); sustained STAT3 recruits DNMT1 and EZH2 (Premise 3); the IRF8 locus acquires dual-mark silencing under these conditions (Premise 4); the bistable switch settles in the off configuration (Premise 2); cDC1 instruction collapses because IRF8 is required for IL-12 production and cDC1 survival (Premise 1) (**Figure 1**). The chain is: SASP → STAT3 → DNMT/EZH2 → IRF8 → cDC1 instruction failure. The lesion operates at two reinforcing levels: at commitment, where progenitors exposed to the SASP-STAT3 field fail to switch the IRF8 enhancer on, so fewer surveillance-competent cDC1s are produced; and at maintenance, where differentiated cDC1s acquire the repressive marks directly observed in tolerogenic, aged, and tumor-bearing states. The chain is minimal in the sense that removing any step breaks the connection between the upstream cause, senescent cell accumulation, and the downstream effect, loss of cDC1 instruction. Each step is independently supported and independently falsifiable. None of the individual steps is novel. Their convergence on cDC1 failure as a single mechanism, applicable across aging, cancer, and chronic infection, is the proposed framework, advanced as the most parsimonious explanation of the shared phenotype rather than as the only possible one.

The framework is parsimonious in the sense that it explains three apparently distinct clinical phenomena as one molecular failure, and it is testable because each step is individually falsifiable. If any premise is wrong, the framework is wrong. The chain also explains a clinical observation that has otherwise lacked a mechanistic account: why senolytic agents (62, 63) produce inconsistent and frequently modest effects on immune function despite efficacy at clearing senescent cells. If the silencing chain’s endpoint, methylated IRF8 with a bistable architecture biased toward off, is installed during the period of SASP exposure, removal of the SASP does not by itself restore IRF8 expression. The installed marks persist. Senolytics address the upstream source without addressing the downstream installation, and the framework predicts this pattern *a priori*.

## 3 The α-DC1 correction

The convergent chain implies a specific therapeutic constraint. Any correction that leaves the upstream SASP field intact and attempts to unlock IRF8 *in situ* must overcome both DNA methylation and H3K27me3 on a bistable locus whose low-affinity enhancer architecture is biased toward the off state. Pharmacological attempts to do this, DNMT inhibitors, EZH2 inhibitors, JAK/STAT3 inhibitors, IL-6 blockade, have produced partial restoration of dendritic-cell function in preclinical settings but not durable correction (39–42). The logic of the chain explains why: each of these interventions targets a single node while the remaining nodes continue to reinforce silencing. DNMT inhibition without STAT3 suppression allows remethylation; STAT3 suppression without demethylation leaves the installed marks in place; EZH2 inhibition without DNA demethylation reveals a still-methylated promoter.

An alternative approach bypasses the architecture rather than unlocking it. A cDC1 manufactured ex vivo in a STAT3-free environment, with IFN-γ priming and TLR3 engagement, is never exposed to the sustained STAT3 signal required for DNMT and EZH2 recruitment. Its IRF8 locus is not methylated because the conditions that drive methylation are absent from the bioreactor. The α-type-1 polarized dendritic cell (α-DC1) protocol developed by Mailliard, Okada, Kalinski, and colleagues produces precisely such a cell: monocyte-derived dendritic cells matured in IFN-γ, IFN-α, poly-I:C, IL-1β, and TNF-α produce markedly higher concentrations of IL-12p70 than conventionally matured dendritic cells (43–45). This IL-12p70 output is not a pharmacological quirk of the maturation cocktail; it is the expected behavior of an IRF8-competent cDC1-equivalent manufactured outside the silencing chain.

Here the α-DC1 is defined as a functional instructor cell, monocyte-derived and matured outside the STAT3-dominated environment, that recapitulates the cDC1 IL-12p70 and cross-priming program. It is advanced on the basis of functional equivalence, not as a developmental cDC1, and the framework does not require lineage identity.

The α-DC1 protocol has been evaluated clinically, most directly in recurrent malignant glioma (45), within a broader body of dendritic-cell vaccine trials in melanoma and other solid tumors (46–48). The clinical data in these trials are consistent with the framework.

Patients who received dendritic-cell-based cancer vaccines produced antigen-specific CD8 T cell responses with IFN-γ secretion, consistent with the α-DC1 retaining the instructional capacity that endogenous tolerogenic dendritic cells have lost.

Objective responses have been documented in a subset of patients. The framework predicts that the strongest responses should occur in those whose baseline immune parameters most closely approximate young-adult levels, a correlation that has not been tested prospectively and that the framework invites.

Interpreted within the framework presented here, the α-DC1 functions as a replacement instructor for a cell population whose native equivalents have been silenced by the mechanism described in Section 2.

**Figure 2:**
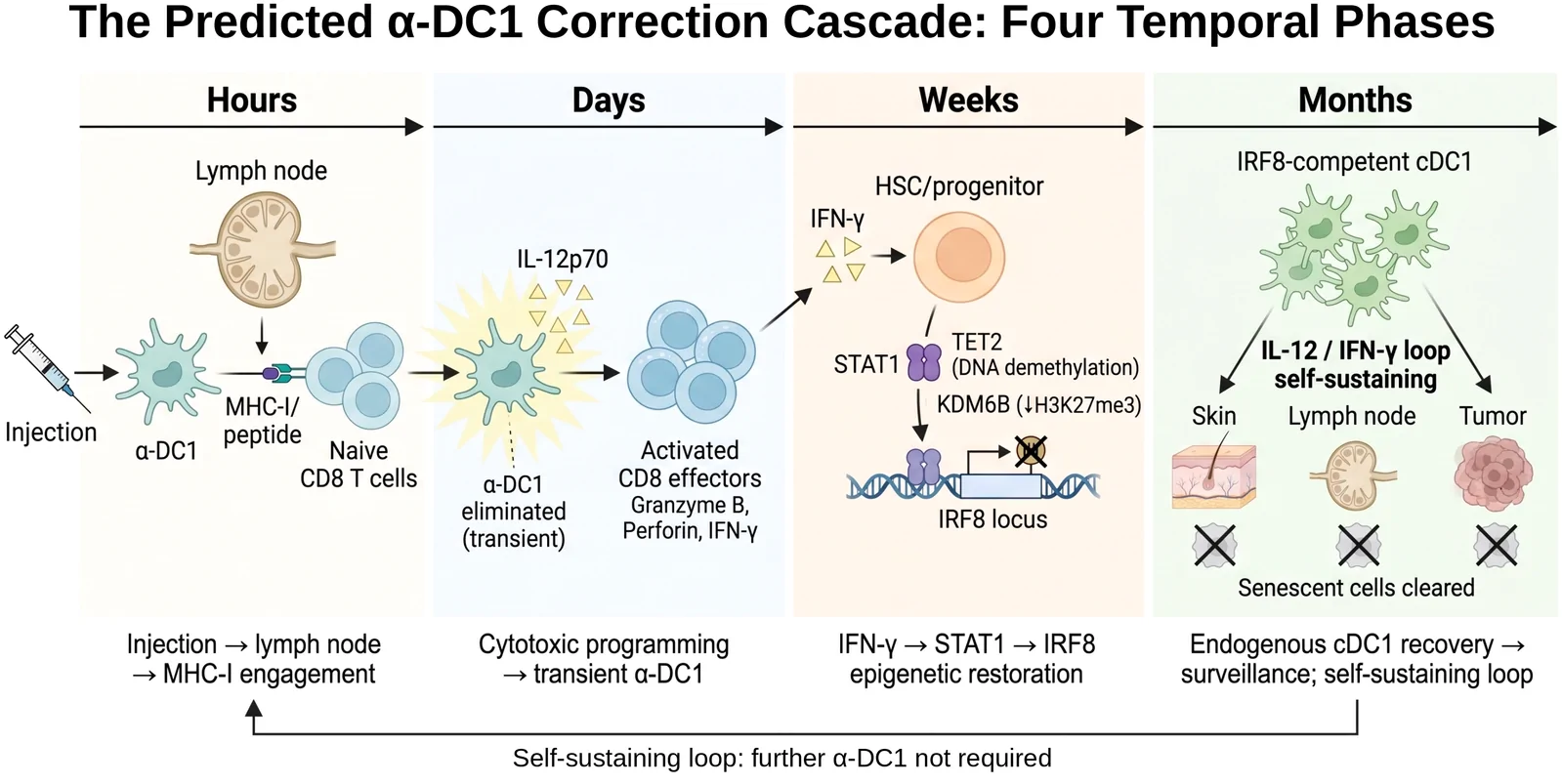
The predicted α-DC1 correction cascade. Four temporal phases of the α-DC1 correction cascade: (1) hours, injection, lymph node drainage, T cell engagement; (2) days, pulsatile IL-12p70 burst, cytotoxic T cell programming, α-DC1 elimination by its own educated effectors; (3) weeks, IFN-γ-driven STAT1/TET2 demethylation and KDM6B-mediated H3K27me3 removal at endogenous IRF8; (4) months, endogenous cDC1 recovery, senescent cell clearance, self-sustaining IL-12/IFN-γ loop. A return arc indicates that once the loop is established, further α-DC1 administration is not required.

The framework predicts a four-phase cascade with distinct temporal signatures (**Figure 2**): (1) hours, injection, lymph node drainage, and MHC class I-mediated engagement of naive CD8 T cells; (2) days, pulsatile IL-12p70 burst, cytotoxic T cell programming (granzyme B, perforin, IFN-γ), and predicted elimination of the α-DC1 by the cytotoxic effectors it educates, a feedback step expected to produce a self-limiting signal rather than chronic exposure. This self-elimination mimics the pulsatile signaling pattern of young-adult homeostasis rather than substituting one chronic signal for another. (3) weeks, IFN-γ secreted by educated T cells activates STAT1, which recruits TET2 to methylated target loci as demonstrated by Peng and colleagues for IRF1 and applicable by mechanism to other IRF-family loci including IRF8 (49, 72); KDM6B (JMJD3), induced in macrophages by inflammatory stimuli in an NF-κB-dependent manner, removes H3K27me3 at inflammatory gene promoters (50). The dual mark is reversed. This is the step of the cascade that converts an ex vivo intervention into an *in vivo* correction: the α-DC1 is eliminated long before this phase, but the IFN-γ field it established continues to propagate through educated effector cells. (4) months, endogenous IRF8-competent cDC1s emerge from progenitors whose loci have now been demethylated, resume senescent cell surveillance, and eliminate the SASP sources that drove the silencing chain in the first place (51). The loop becomes self-sustaining: IRF8-competent cDC1s produce IL-12, IL-12 drives IFN-γ, IFN-γ maintains STAT1/TET2/KDM6B activity, and further α-DC1 administration is not required. The framework thus reframes the α-DC1 from a cancer vaccine to a biological senolytic: its function is not to kill senescent cells directly, as pharmacological senolytics do, but to restore the endogenous immune capacity to clear them.

## 4 Testable predictions

The framework is useful only to the extent that it makes predictions distinguishable from existing models of immune aging and dendritic cell dysfunction. We offer four, each with an explicit falsification criterion. Each prediction is independently testable using clinical or laboratory methods already in routine use. If any of them fails in appropriately designed studies, the framework requires revision or abandonment. We emphasize that the predictions are intended as falsification conditions, not as claims. Until they are tested, the framework remains a hypothesis.

## 5 Discussion and limitations

We have proposed that a single four-step molecular chain explains three apparently distinct clinical phenomena, immune aging, cancer immune evasion, and chronic infection tolerance, and that a cell therapy already in clinical use bypasses the chain by construction rather than by pharmacology. Several limitations bear on the strength of this proposal.

Several mechanisms that operate across aging, cancer, and chronic infection are best understood as inputs to or consequences of the proposed chain rather than as alternatives to it. IL-10 is a canonical activator of STAT3, and the IL-10 to STAT3 to DNMT to IRF8 silencing sequence has been demonstrated directly, with IL-10-activated STAT3 binding the Dnmt1 and Dnmt3b promoters and driving IRF8 promoter hypermethylation (73), and the same axis sustains myeloid-derived suppressor cells through autocrine IL-6 (77). Transforming growth factor beta, type I interferon, metabolic suppression, and persistent antigen each sustain the chronic STAT3 state placed at Premise 5; altered progenitor output enters at Premise 5 through inflamm-aging of hematopoiesis; and T cell exhaustion is a downstream consequence of IL-12 loss. The principal independent variable is the tissue-specific dendritic-cell niche. The convergence proposed here is therefore bounded: it identifies a shared, correctable node into which these context-specific signals feed, not a claim that one pathway acts in isolation.

First, α-ketoglutarate/TET competition, one candidate route for the reversal arm of the cascade, is established in other cell types but has not been directly demonstrated in cDC1 progenitors specifically. It remains a prediction rather than a confirmed observation. The framework is compatible with alternative demethylation pathways, including active demethylation by STAT1-recruited TET2 without metabolic modulation, but the quantitative contribution of each pathway to IRF8 restoration following α-DC1 vaccination is unknown.

Second, the SASP is heterogeneous across tissues and disease states, and the specific cytokine dominance that sustains STAT3 may differ between aging, cancer, and chronic infection settings. The framework treats these as variations on a theme, but the quantitative contributions of individual SASP components in each setting remain to be mapped. Whether IL-6 trans-signaling is the dominant driver in all three contexts, or whether context-specific cytokines (TGF-β in tumors, type I IFN in chronic viral infection) play comparable roles, is an empirical question the framework does not resolve.

Third, the bistable architecture at IRF8 is best characterized in murine cDC1s. The human enhancer topology may differ in quantitative detail, though the binary behavior of the human locus appears preserved based on available bisulfite sequencing and chromatin accessibility data (11, 12, 25). Extrapolation from mouse to human remains a standard limitation of lineage-tracing and enhancer- architecture literature in dendritic cell biology.

Fourth, the framework predicts durable correction from a pulsatile intervention. This prediction depends on the loop becoming self-sustaining after α-DC1 elimination. If the loop is not self-sustaining, if, for example, the SASP sources regenerate faster than endogenous cDC1s clear them, booster vaccinations may be required, and the translational profile of the α-DC1 would more closely resemble a conventional vaccine than a one-time biological correction. The predicted DNA-methylation aging trajectory (57, 58) read out by the pace-of-aging measure DunedinPACE distinguishes these scenarios: a self-sustaining loop predicts continued deceleration between 6 and 24 months, while a non- self-sustaining loop predicts deceleration only during the period of active intervention.

Fifth, the IFN-γ→STAT1→TET2 recruitment mechanism central to the Phase 3 cascade has been directly demonstrated at the IRF1 locus in renal cell carcinoma (72). Extrapolation to IRF8 in cDC1 progenitors is mechanistically reasonable, IRF1 and IRF8 are paralogous transcription factors with analogous promoter architectures, but the specific demonstration of TET2 recruitment to IRF8 by IFN-γ-activated STAT1 in dendritic cell progenitors remains to be performed. This is one of the direct experimental tests the framework invites.

The framework makes no claim to novelty at the level of individual molecular observations. IRF8 biology, STAT3-DNMT interactions, SASP characterization, and α-DC1 manufacturing are all established. What is proposed is the assembly of these observations into a single convergent chain and the recognition that the α-DC1 protocol, developed for oncology indications, is the specific corrective for a mechanism that extends beyond oncology to aging and chronic infection. If the framework is correct, the α-DC1 is a biological senolytic that restores the endogenous capacity to clear senescent cells rather than a chemical senolytic that eliminates them directly. The distinction is therapeutically consequential: biological senolytic activity operates through the native immune system and is subject to its regulatory checkpoints, while chemical senolytic activity bypasses those checkpoints and may produce collateral effects on non-senescent cells.

A further concern is that the framework depends on increased IFN-γ from educated CD8 T cells, whereas aged T cells already overproduce IFN-γ as part of inflammaging (84). The two signals are distinct. The tonic IFN-γ of the aged compartment is antigen-nonspecific bystander output from a dysregulated T cell pool, and that disordered production is itself part of the lesion (84). The IFN-γ in this framework is the opposite: antigen-specific, cDC1-licensed, target-directed, and pulsatile, produced within a coordinated type-1 response that self-terminates as the α-DC1 is eliminated. The framework therefore predicts that correction lowers the inflammatory drive rather than raising it, because restored cDC1 surveillance clears the senescent cells that are the SASP sources of the tonic signal.

We close by noting that the implications of the chain, if correct, extend considerably beyond the scope of this paper. A single molecular failure that can be bypassed ex vivo by a cell therapy already in clinical use has consequences that merit systematic investigation rather than advocacy. The same loss of senescence surveillance is increasingly implicated in neurodegeneration, where clearing senescent glial cells prevents tau-dependent pathology and cognitive decline (55), cellular senescence contributes to the neuropathology of Parkinson disease (64), and senescent-cell burden is tied to Alzheimer disease (69); whether the cDC1 instruction axis participates in these settings is an open question the framework raises but does not resolve. The most direct falsification is the proximal one specified in Prediction 1, reversal of IRF8 methylation in peripheral cDC1s following α-DC1 vaccination, together with the recovery of IL-12p70 capacity in Prediction 2. The most consequential prediction is the DunedinPACE measurement at 12 months. Until these measurements are made, the framework remains a hypothesis.

### 5.1 Retention of α-DC1 function after transfer

A reasonable concern is whether α-DC1 retain Type-1 function after transfer into an IL-6-rich, STAT3-promoting environment. Three considerations bear on this. First, α-DC1 are terminally Type-1 polarized through maturation in interferon gamma, interferon alpha, polyinosinic:polycytidylic acid, interleukin-1 beta, and tumor necrosis factor alpha, and are committed rather than plastic. Second, the corrective signal is pulsatile and self-limiting, because α-DC1 are eliminated within days by the effectors they prime, so prolonged residence in an IL-6-rich field is not required; this is the same distinction between transient and sustained STAT3 signaling drawn at Premises 3 and 5, since it is sustained STAT3 that installs the silencing marks. Third, where additional protection is desired, the product can be rendered STAT3-resistant by transient STAT3-degrader preconditioning, enforced SOCS3 expression, or short-course IL-6 or IL-6 receptor blockade bracketing transfer; STAT3 in dendritic cells is druggable, and its degradation reprograms dendritic cells toward immunogenicity *in vivo* (74). Committed DC1 precursors generated through sequential STAT3 and STAT5 activation resist many tumor-associated immunosuppressants and produce IL-12p70 on tumor contact (76). A confirmatory assay measuring α-DC1 IL-12p70 output and phosphorylated STAT3 after exposure to aged serum or IL-6, with and without these interventions, is proposed.

### 5.2 Context-specific predictions

The lesion proposed as conserved across the three contexts is upstream: a STAT3-dominated environment that silences IRF8 and collapses cDC1 IL-12p70 licensing capacity. The responding CD8 T cell differs by context, comparatively naive but declining in aging and driven toward exhaustion in cancer and chronic infection. In cancer, α-DC1 re-licensing should expand cytotoxic T lymphocytes against defined tumor antigens and should synergize with checkpoint blockade in exhaustion-dominated tumors. In chronic viral infection, α-DC1 should restore antigen-specific CD8 responses and reduce inhibitory-receptor expression, with benefit tracking the reversibility of the exhausted state. Restoration of the IRF8 node is achievable *in vivo*: delivery of IRF8 into established tumors reprograms intratumoral myeloid-derived suppressor cells into antigen-presenting cells, enriches cDC1 and CD8 T cells, lowers Arg1 and IDO1, and prolongs survival (78), a proof-of-concept for the node distinct from the α-DC1 route. The framework concerns bioactive IL-12p70 specifically, not bulk IL-12p40, which is shared with IL-23 and is not the licensing species.

This distinction predicts a dissociation rather than a deficiency. IL-12 is produced by inflammatory monocytes, monocyte-derived dendritic cells, and tissue macrophages as well as by cDC1 (85, 86), and the bioactive instruction depends on its timing rather than on abundance (87). The framework therefore predicts that bulk IL-12, and especially the shared p40 subunit, is preserved or elevated across aging, cancer, and chronic infection while the cDC1-licensed, pulsatile IL-12p70 instruction is selectively lost. This is the same inflammation-amid-immunodeficiency pattern drawn for IFN-γ, where tonic dysregulated cytokine coexists with failed antigen-specific Type-1 immunity, and it clarifies the correction: the α-DC1 does not raise an ambient cytokine level that is not deficient, but supplies a licensed, time-limited IL-12p70 signal from a cell built outside the silencing field, a qualitatively different input rather than more of the same. The prediction is falsifiable: if cDC1-specific IL-12p70 output and cross-priming capacity are intact in aged or diseased subjects relative to young or healthy controls, or if the functional deficit tracks bulk p40 rather than cDC1 p70, the dissociation claim fails.

### 5.3 The STAT4/IL-12 reversal mechanism

The correction proposed here has a specific molecular basis. The cascade installs the silencing through STAT3 activating the writers directly: IL-10-activated STAT3 binds the Dnmt1 and Dnmt3b promoters and drives IRF8 promoter hypermethylation in myeloid-derived suppressor cells (73). The licensed IL-12p70 the α-DC1 delivers opposes this through STAT4. IL-12 through STAT4 remodels the IRF8 locus toward activation. In activated lymphocytes, chromatin immunoprecipitation sequencing places STAT4 on the IRF8 promoter at a region of open chromatin, with IRF8 among the most highly STAT4-bound loci, and the activating H3K4me3 mark at the IRF8 locus is STAT4-dependent (88; reviewed in 94). The chromatin program STAT4 drives is the inverse of the silencing arm: it establishes H3K4 methylation, recruits the demethylase Jmjd3, and produces a Jmjd3-dependent loss of both H3K27me3 and DNMT3a association at STAT4 target loci in mice (90), removing the EZH2-installed mark and its writer together. At the IRF8 promoter specifically, H3K4me3 enrichment overrides complete CpG methylation and permits transcription in myeloid cells (89), so activation does not require demethylation to finish first. IL-12 further promotes TET2-dependent demethylation of existing marks (91), and IL-12 production in the central nervous system upregulates SOCS3, the dedicated STAT3 inhibitor, within the infiltrating mononuclear population, with STAT4 itself activated and nuclear-localized (92), restoring the brake the cascade had removed. Human microglia express both IL-12 receptor subunits, activate STAT4 with nuclear translocation, and produce bioactive IL-12p70 themselves in response to IL-12 stimulation (93), closing the self-sustaining loop the framework predicts. STAT4 and STAT3 compete for the same chromatin targets; the α-DC1 delivers the STAT4 signal from outside the inflammatory field where STAT3 dominates.
